# Anthropometric nutritional status of children (0–18 years) in South Africa 1997–2022: a systematic review and meta-analysis

**DOI:** 10.1017/S1368980023001994

**Published:** 2023-11

**Authors:** Herculina Salome Kruger, Marina Visser, Linda Malan, Lizelle Zandberg, Mariaan Wicks, Cristian Ricci, Mieke Faber

**Affiliations:** 1 Centre of Excellence for Nutrition, North-West University, Potchefstroom, 2520 South Africa; 2 Medical Research Council Unit for Hypertension and Cardiovascular Disease, North-West University, South Africa; 3 Africa Unit for Transdisciplinary Health Research, North-West University, Potchefstroom, South Africa; 4 Non-Communicable Diseases Research Unit, South African Medical Research Council, Cape Town, South Africa

**Keywords:** Obesity, Stunting, Nutritional status, Africa

## Abstract

**Objective:**

To conduct a comprehensive systematic review and meta-analysis of the available literature on the anthropometric nutritional status of South African infants and children, 0–18 years old and to report on trends of changes in nutritional status over the period 1997–2022.

**Design:**

Systematic review and meta-analysis.

**Setting:**

Review of the available literature on the anthropometric nutritional status of South African infants and children, 0–18 years old, over the period 1997–2022.

**Participants:**

South African infants and children, 0–18 years old.

**Results:**

Only quantitative data from ninety-five publications that described the nutritional status in terms of anthropometry were included. Most recent studies applied the WHO 2006 and 2007 definitions for malnutrition among children 0–5 years old and 5–19 years old, respectively. Meta-analysis of all prevalence data shows the highest stunting prevalence of 25·1 % among infants and preschool children, compared to 11·3 % among primary school-age children and 9·6 % among adolescents. Furthermore, the overweight and obesity prevalence was similar among children younger than 6 years and adolescents (19 %), compared to 12·5 % among primary school-age children. In national surveys, adolescent overweight prevalence increased from 16·9 % in 2002 to 23·1 % in 2011. Meta-regression analysis shows a decrease in stunting among children 6–18 years old and an increase in combined overweight and obesity in the 10–19 years age group.

**Conclusion:**

The double burden of malnutrition remains evident in South Africa with stunting and overweight/obesity the most prevalent forms of malnutrition among children.

## Introduction and rationale

Children represents a large proportion of the population of South Africa, with 28·1 % of the population younger than 15 years^(1)^. Early investment in child health is important to promote optimal growth and development and will contribute to a healthy and productive adult workforce. The first 1000 days of life as well as adolescence are critical windows of development, determining susceptibility to adult obesity and cardiometabolic health. Environmental insults during these rapid development phases may result in irreversible adverse outcomes^(2)^. Optimal nutrition in school-age children promote physical and mental development and contribute to social and economic development^(3)^. A study of first-grade South African learners showed that even moderate stunting and wasting among children were associated with suboptimal school performance and motor function skills^(4)^.

In the most recent national survey, stunting (27·4 %) and combined overweight and obesity (13·3 %) were the most prevalent forms of malnutrition among children younger than 5 years of age in South Africa^(5)^. Smaller proportions of preschool children were underweight (5·9 %) and wasted (2·6 %). Among adolescents aged 15 years and older, 6·7 % of girls and 20·7 % of boys were underweight, 15·8 % of girls and 6·1 % of boys were overweight, while 11 % of girls and 2·5 % of boys were obese^(5)^. The highest prevalence of stunting is generally found among children younger than 5 years, but stunting has also been reported among school-age children^(6)^ and up to late adolescence^(7)^. Besides these national surveys, several smaller studies contribute important information about the regional differences in nutritional status of South African children. Such information is important to inform policies and programmes in countries with limited resources so that the most vulnerable groups and provinces can be targeted.

The aim of this study was to conduct a comprehensive systematic review and meta-analysis of the available literature on the anthropometric nutritional status of South African infants and children of 0–18 years old and to report on trends of improvement or deterioration in nutritional status over the period 1997–2022.

### Protocol

The protocol was drafted using the Preferred Reporting Items for Systematic Reviews and Meta-analysis Protocols (PRISMAP) and was revised by the research team. The protocol was not registered at PROSPERO, since no health-related outcomes were included in this review. The review was strictly not a scoping review to identify gaps in the available literature, but a systematic review of prevalence studies.

## Methods

### Eligibility criteria for study selection

Observational cross-sectional studies as well as the baseline data of randomised controlled trials or prospective studies published in English after 1996 on the nutritional status of South African children were included in this systematic review. For some cohort studies with a baseline before 1997, the most complete data of a year between 1997 and 2022 were included. The inclusion criteria were the following: healthy South African children, 0–18 years old, and original quantitative data on assessment of anthropometric nutritional status. Data from some studies included adolescents or high school children up to the age of 19 years. Data for these 19-year-old adolescents were included in the grouped data of adolescents. Studies reporting quantitative estimates of nutritional status of South African children, such as prevalence of malnutrition, were included. Studies were excluded if they were intervention studies or clinical studies in patient subgroups, young pregnant or lactating women and/or particularly vulnerable groups, such as orphans. However, studies in large groups from low socio-economic status were included, because a large proportion of South African children live in low socio-economic households. Narrative and systematic reviews, letters, editorials, case–control and qualitative studies as well as studies with data collection before 1997 were excluded. Most of the latter were included in a previous review of the nutritional status of South African children^(8)^.

### Search strategy

Literature searches were performed in PubMed, Ebscohost CAB Abstracts, CINAHL and the African Journals databases for the period 1 January1997 to 31 July 2022, by using a structured search strategy based on the eligibility criteria. The search strategies were drafted by an experienced librarian and further refined through team discussion. Relevant keywords were identified from the Medical Subject Headings (MeSH) terms and adapted for each database. The search syntax for PubMed, Ebscohost and CINAHL is shown in Table 1. The syntax was modified for the African Journals database, with ‘nutrition’ in ‘Anywhere’ and filters for ‘Medicine and Health’, SciELO SA and the start and ending dates, due to the limited options in the advanced search. We used an iterative process to identify appropriate search terms, including a term regarding nutritional status, malnutrition (undernutrition, underweight, stunting, wasting, overweight or obesity) and children (terms for the different age groups). We also included ‘South Africa’ and date of publication in the search string. No grey literature was included, because most studies from South African students’ dissertations are published in South African scientific journals. Furthermore, unpublished dissertations would probably not achieve the required quality score for inclusion in this review.

**Table 1:** Search terms for the PubMed, literature search

#1:	All fields/All text fields	‘Nutritional status’ OR malnutrition OR undernutrition OR underweight OR wasting OR stunting OR overweight OR obesity
AND	Title/Abstract	‘South Africa’ OR ‘sub-Sahara Africa’
AND	Title/Abstract/Abstract	Infant OR baby OR child OR pediatric OR paediatric OR ‘young child’ OR toddler OR pre-school OR preschool OR ‘early childhood’ OR adolescent OR teenager OR youth
AND	Date – Publication/Publication year	1997/01/01 to 2022/07/31 1997 to 2022

### Title, abstract and full-text screening and quality assessment

Titles and abstracts retrieved from electronic searches were screened by two independent reviewers (HSK and MVV) after initial removal of duplicates. If the two reviewers could not agree on inclusion, they consulted with a third reviewer (MF) and made a final decision based on consensus. Eligible studies were selected based on the inclusion and exclusion criteria. Finally, full-text articles were screened and reasons for exclusion were noted. Reviews were excluded, but additional studies were identified from the reference lists of systematic and narrative reviews. Eligible studies were further screened by two independent reviewers (HSK and MVV) for the assessment of the quality of the reported data, based on the Joanna Briggs Institute critical appraisal scoring system for studies reporting prevalence data proposed by Munn *et al.* ^(9)^ The reviewers scored the studies with a ‘Yes’ answer to each question to receive a score of 1, while a ‘No’ answer received a score of 0, with a maximum score of 9. A minimum total score of 5 was used as the threshold for the final inclusion of a study into the systematic review, but studies with a score above 5 were excluded if incorrect cut points for child anthropometric nutritional status were applied.

### Data extraction and synthesis

**Fig. 1:**
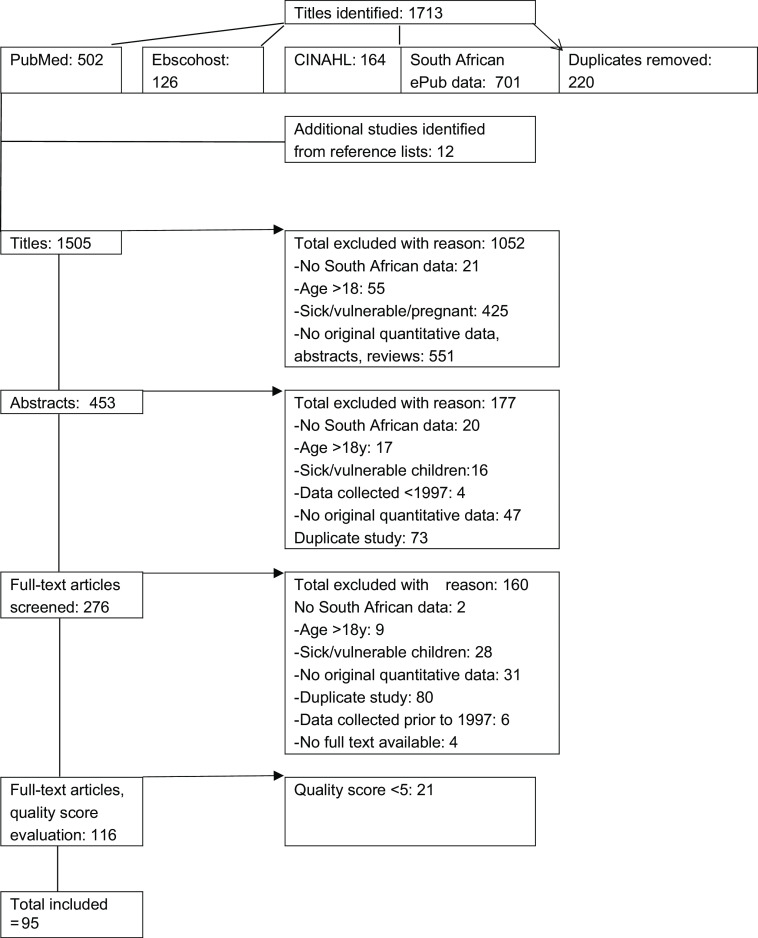
PRISMA flow diagram of the screening procedure followed to identify eligible studies

A flow chart showing the number of studies assessed and included in this review is shown in Fig. 1. A data extraction form was developed by the review team based on the objectives of the review. Two reviewers piloted the form (HSK and MVV) and added columns to include important information. One reviewer used the final form for recording the data extracted from each of the eligible studies (HSK). A second reviewer (MVV) checked each set of extracted data, and in case of differences, the data were discussed with a third reviewer (MF). The following information was extracted from all eligible articles: (a) first author’s surname and publication date; (b) year of study; (c) province where the study was conducted; (d) the study setting and location (rural or urban); (e) participants’ age range; (f) representativeness of the sample; (g) sample size; (h) reference used to indicate anthropometric nutritional status, that is, WHO 2006 for infants and children aged 0–5 years, WHO 2007 for children aged 5–19 years, Center of Disease Control (CDC), National Center of Health Statistics (NCHS) or International Obesity Task Force (IOTF) cut points for overweight and obesity; (i) mean ± standard deviation or median and IQR of anthropometric nutritional status marker, and (j) prevalence of nutritional status category (%). Data are presented according to three age groups: infants and preschool children (0–6 years); primary school-age children (6–13 years) and adolescents (12–18 years). When the data were reported according to overlapping age groups, for example, 10–14 years, the data were presented in the category representing most of the children. Most studies were represented by a single article, but in a small number of cases, the required data were available from more than one article, for example, studies presenting different anthropometric data stratified in different articles.

The data were synthesised based on the different objectives, and data from studies were summarised. We grouped studies according to age category, different forms of malnutrition (stunting, underweight, wasting, overweight and obesity) and province where the data were collected. The prevalence of different nutritional status indicators per age category over time were compared to note any improvements or deterioration in nutritional status markers over the period of study (1997–2022).

### Statistical methods

In the assessment of the quality of the included publications, agreement between the scores of two independent reviewers was assessed by calculating the kappa statistic and the intra-class correlation for single measures. The meta-analysis of prevalence was conducted using a random effects model only for studies that used the same references and cut points. Briefly, in such a model the study’s weight was the inverse variance with the weight of the i-th study, computed as w_i_ = 1/(si^2^ + t^2^), where si^2^ was the variance of the i-th study, and t^2^ was the overall variance. The Cochrane Q test and the I^2^ statistic were reported to show the between-study heterogeneity. Publication bias was investigated using the Egger test^(10)^.

A random effect meta-regression was conducted to identify the source of between-study heterogeneity, and the between-study heterogeneity accounted by the meta-regression was performed by the residual I-squared^(11)^. Factors included in the meta-regression model included year of data collection, rural *v*. urban area, quality score according to the Joanna Briggs Institute scoring system and sampling method (convenience *v*. random). Data were transformed using the arc sin function, and results were provided after retro transformation of results performed using the sin function. All statistical analyses were performed using the STATA software version 14. The Forest plots were performed by a custom Excel macro, and the metan and the metareg function of STATA were used to conduct the meta-analyses and meta-regression, respectively. All statistical tests were conducted with a significance level of 5 % (*α* = 0·05), except for the Egger test where a significance level of 10 % (*α* = 0·1) is recommended^(10)^.

## Results

In total, 1505 titles, 453 abstracts and 276 full-text articles were screened for inclusion (Fig. 1). Of these, 116 full-text articles were assessed in terms of quality, which yielded the final number of ninety-five studies. The kappa statistic for agreement between the scores of two independent reviewers was 0·47, *P* < 0·0001, and the intra-class correlation for single measures was 0·94 (95 % CI = 0·91, 0·96). Kappa was interpreted using 0·41–0·60 as moderate agreement and > 0·60 as very good agreement^(12)^. Intra-class correlation coefficients were interpreted as 0·50–0·75 as moderate and > 0·75 as good agreement^(13)^.

### Description of studies, study participants and methods used to assess nutritional status

The studies included in this systematic review presented data on a total of ninety-five studies, including national surveys^(5–7)^, and from all nine provinces of South Africa, with the sample sizes within individual studies ranging from 31 to 10 195 participants.

The most commonly used reference to define anthropometric nutritional status was the NCHS cut points in publications up to year 2010^(14)^. The IOTF cut points to define overweight and obesity were used often since 2005^(15)^, while most recent studies applied the WHO 2006 definitions for malnutrition among children 0–5 years old and the WHO 2007 definitions for children 5–19 years old^(16,17)^, while the CDC cut points and the recent IOTF cut points for thinness were also used^(18)^.

### Anthropometric nutritional status

Most regional studies in preschool children (0–6 years) were conducted in rural areas (68 %), while a similar proportion of studies among school-age children were from rural and urban areas. Race and ethnicity of the study participants were not always reported, but only two studies in children from one race were included in this review^(19,20)^. National studies and studies with a large sample size generally included children from all race groups.

#### Infants and preschool children

**Fig. 2:**
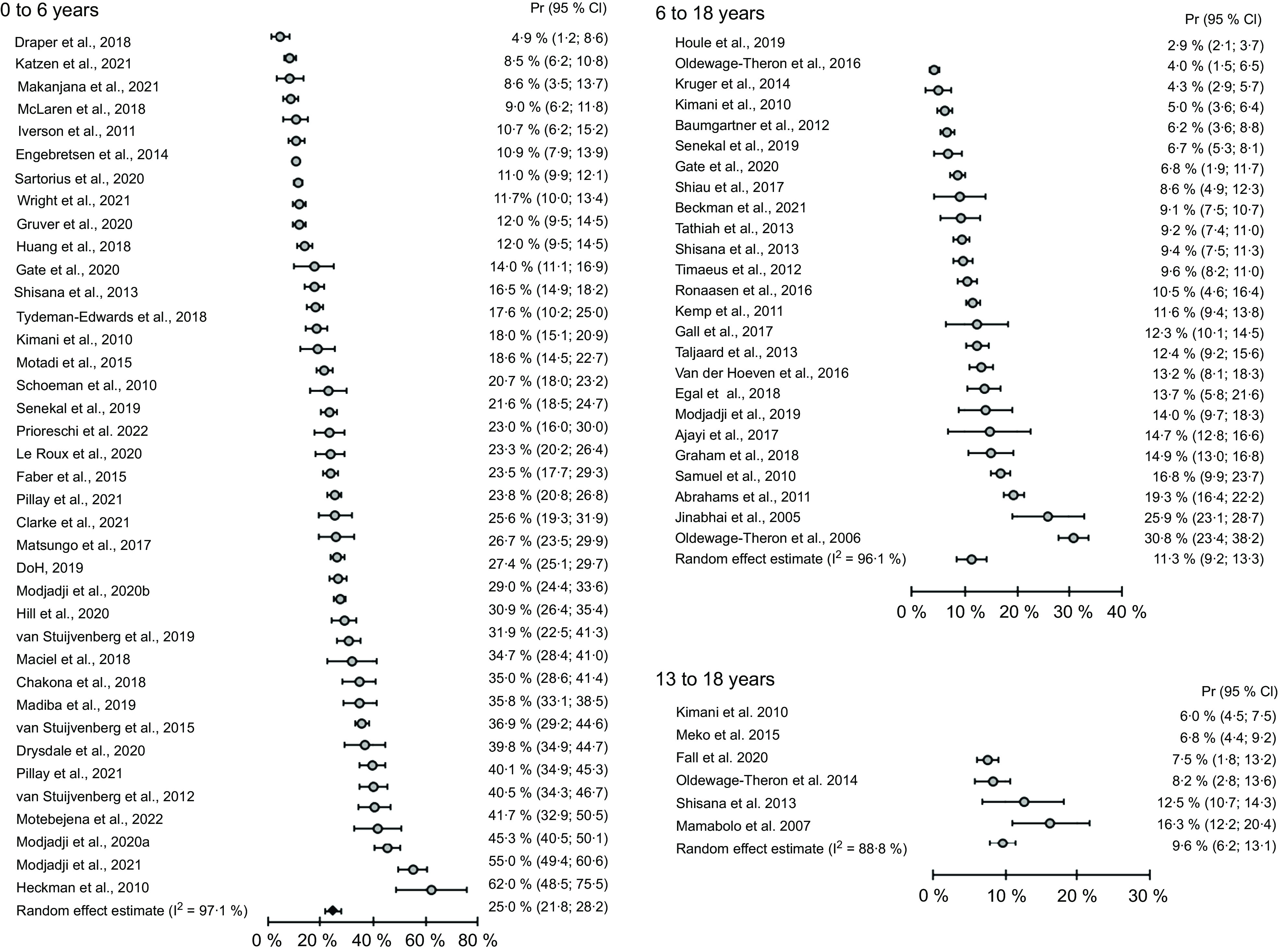
Meta-analysis of stunting prevalence data of South African preschool and primary school-age children and adolescents based on studies published from 2006 to 2022. Pr, prevalence; stunting classified according to the WHO Growth Standard 2006^(14)^ and Growth Reference data 2007^(15)^

**Fig. 3:**
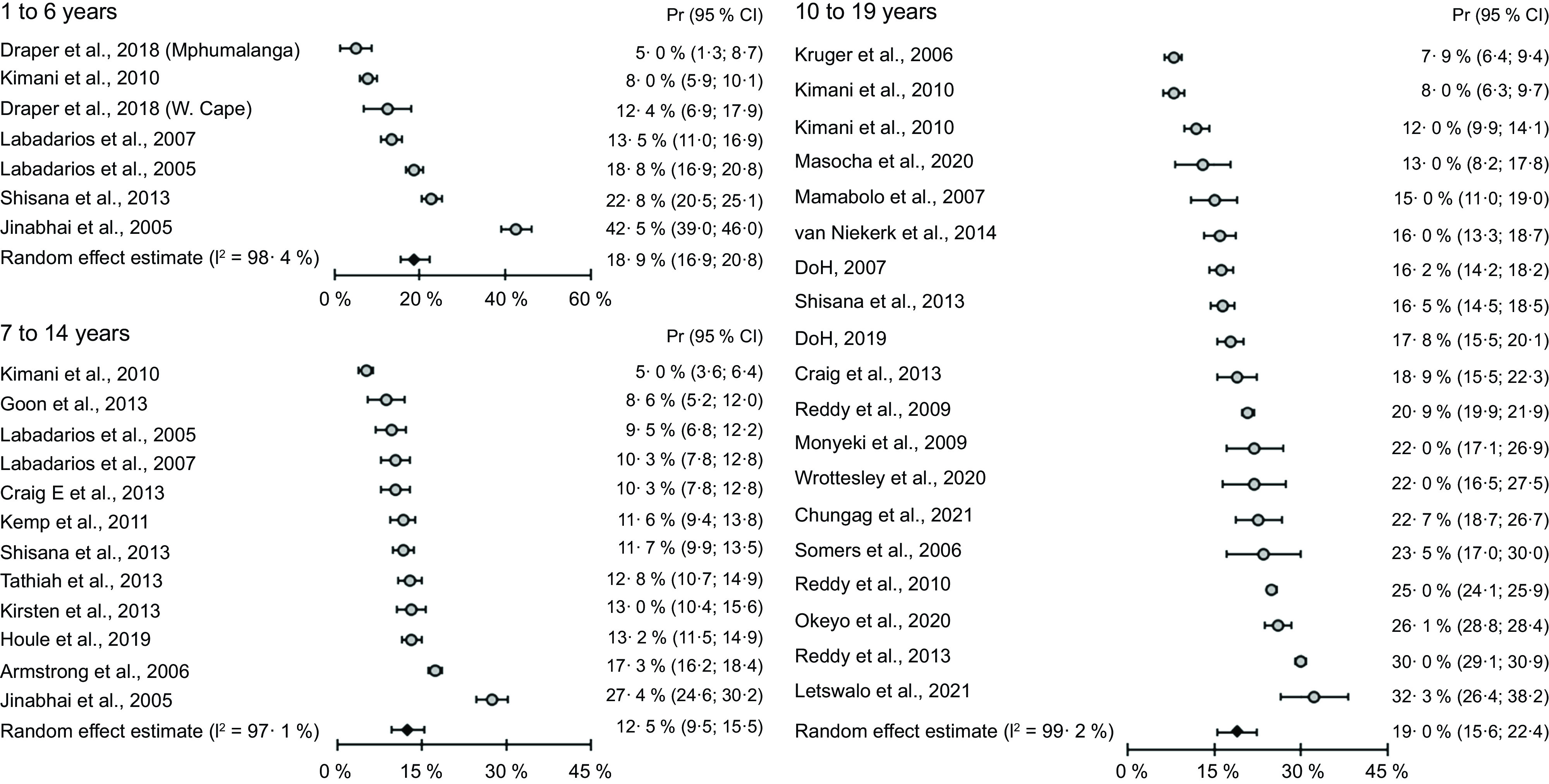
Meta-analysis of prevalence data of combined overweight and obesity among South African preschool and primary school-age children and adolescents based on studies published from 2005 to 2021. Pr, prevalence; overweight and obesity classified according to the International Obesity Task Force cut points proposed by Cole et al., 2000^(13)^

The prevalence of stunting, overweight and obesity among preschool children from national studies and selected regional studies with a large sample size is presented in Table 2, and complete data are presented in Table S1. Forest plots of the prevalence of stunting as well as combined overweight and obesity among infants and preschool children are presented in Figs 2 and 3, respectively. Meta-analysis of all prevalence data shows a stunting prevalence of 25·0 % among infants and preschool children. Generally, a higher prevalence of stunting was reported in rural settings (18–37 %)^(21–27)^ than in urban areas (1·8–10·9 %)^(19,28,29)^, except for two studies that reported high prevalence of stunting in low socio-economic settings in urban areas (19–36 %)^(30–32)^. In regional, as well as in national studies, for the total under 5 years old group, the highest prevalence of stunting, underweight and wasting was found in children from the Northern Cape province^(33–35)^. A persistent high prevalence of stunting was reported among children younger than 5 years of age in nationally representative studies over time since 1999^(5,35–37)^. The highest prevalence of underweight and wasting was found in studies where particularly low socio-economic groups were targeted^(32–34)^, while a relatively low prevalence of underweight and wasting in preschool children were reported in most other studies. In nationally representative studies, the prevalence of underweight and wasting apparently decreased from 1999 to 2016^(5,35)^. The most recent national survey showed that the highest prevalence of under-five stunting was found in the age range 18–23 months and among children from the poorest households^(5)^. The meta-regression analysis showed no change in stunting prevalence in this age group over the study period (*P* = 0·23, Table 3).

**Table 2:** The prevalence of stunting, underweight, overweight and obesity among preschool children from national studies and selected regional studies with a large sample size by province and reference

Province study, setting	Year of study	Age group	Sample size	Stunting (%)	Underweight (%)	Overweight/obese	Cut-point reference	Prevalence (%, (95 %CI)) All, M/F		Reference
Reference: NCHS HAZ < –2; WHO 2006 WAZ < –2 WHZ > 2/ > 85th and 95th percentiles										
National	1999	1–3 years	1198	25·5	12·4	Overweight	NCHS WHZ > 2	6·6	(5·2, 8·0)	Labadarios *et al.*, 2005^(35)^
EC R	2003	0·5–2 years	767	10·7	–	Overweight	NCHS WHZ > 2	15·2		Smuts *et al.*, 2008^(65)^
EC R	2003	2–5 years	765	–	–	Overweight	NCHS WHZ > 2	5·0		Smuts *et al.*, 2008^(65)^
Reference: WHO 2006 HAZ < –2; WHZ/BAZ > 2										
National	1999	1–3 years	1160	25·5	12·4	Overweight	WHZ > 2	11·3	(9·3, 13·4)	Labadarios *et al.*, 2005^(35)^
National	1999	4–6 years	993	23·4	11·0	Overweight	WHZ > 2	6·6	(4·6, 8·8)	Labadarios *et al.*, 2005^(35)^
KZN U	2016	4–6 years	627	–	12·0				–	Gruver *et al.* 2020^(66)^
KZN R	2018	0–5 years	567	14·0	1·9	Overweight	WHZ > 2	11·8	11·8	Gate *et al.*, 2020^(62)^
						Obese	WHZ > > 3	3·7		
KZN, NC, WC, Limp	2011	1–6 years	747	23·5	9·8	Overweight	WHZ > 2	6·1		Faber *et al.*, 2015^(67)^
WC R	2012–5	< 1 years	1076	–	–	Overweight	BAZ > 2	9·0		Budree *et al.*, 2017^(68)^
NWP U	2013–5	0·5 years	750	26·7	11·1	Overweight	BAZ > 2	10·1		Matsungo *et al.*, 2017^(30)^
NC U	2017	6 weeks	733	23·3	13·6	Overweight	WHZ > 2	11·0		Le Roux *et al.*, 2020^(69)^
National	2016	1–5 years	1416	27·4	5·9	Overweight	WHZ > 2	13·3		NDoH, StatsSA & ICF, 2019^(5)^
National SA-NIDS	2008	0·5 years	3254	11·0	5·2	Overweight	WHZ > 2	14·5		Sartorius *et al.*, 2020^(70)^
Gauteng RU	2018	0–5 years	674	21·6	5·6	Overweight	BAZ > 2	10·3		Senekal *et al.*, 2019^(42)^
						Obese	BAZ > > 3	7		
Reference: NCHS/WHO 2006 HAZ < –2; International Obesity Task Force (IOTF) cut points										
KZN RU	1998	2–5 years	770	33·7	5·8, 6·8	Overweight	IOTF		27·6 M, 23·9 F	Jinabhai *et al.*, 2005^(71)^
KZN RU	1998	2–5 years	770			Obese	IOTF		13·9 M, 12·4 F	Jinabhai *et al.*, 2005^(71)^
National	1999	1–3 years	795	25·5	12·5	Overweight	IOTF	16·0	(13·7, 18·2)	Labadarios *et al.*, 2005^(35)^
National	1999	1–3 years	795			Obese	IOTF	7·8	(6·1, 9·5)	Labadarios *et al.*, 2005^(35)^
National	1999	4–6 years	861	20·7	8·8	Overweight	IOTF	12·0	(9·6, 14·4)	Labadarios *et al.*, 2005^(35)^
National	1999	4–6 years	861			Obese	IOTF	3·8	(2·5, 5·1)	Labadarios *et al.*, 2005^(35)^
National	2005	1–3 years	846	23·4	11·0	Overweight	IOTF	13·0	(10·8, 15·2)	Labadarios *et al.*, 2007^(36)^
National	2005	1–3 years	846			Obese	IOTF	6·3	(4·7, 7·8)	Labadarios *et al.*, 2007^(36)^
National	2005	4–6 years	745	20·7	8·8	Overweight	IOTF	8·3	(6·2, 10·4)	Labadarios *et al.*, 2007^(36)^
National	2005	4–6 years	745			Obese	IOTF	2·6	(1·4, 3·7)	Labadarios *et al.*, 2007^(36)^
National	2005	1–9 years	2157	18·0		Overweight	IOTF	10·0	(8·7, 11·3)	Labadarios *et al.*, 2007^(36)^
National	2005	1–9 years	2157			Obese	IOTF	4·0	(3·1, 4·8)	Labadarios *et al.*, 2007^(36)^
Mphu R	2007	1–4 years	671	18·0	10·0	Overweight	IOTF	7		Kimani-Murage *et al.*, 2010^(72)^
						Obese		1·0		
National	2012	1–3 years	1090	26·5	6·1	–	–		–	Shisana *et al.*, 2013^(6)^
National	2012	4–6 years	954	11·9	4·5	–	–		–	Shisana *et al.*, 2013^(6)^
National	2012	2–5 years	1291	–	–	Overweight	IOTF	18·1	(11·4, 23·7)	Shisana *et al.*, 2013^(6)^
National	2012	2–5 years	1291	–	–	Obese	IOTF	4·6	(2·2, 7·0)	Shisana *et al.*, 2013^(6)^

U, urban; R, rural; M, male; F, female; NCHS, National Center for Health Statistics; HAZ, height-for-age z-score; WHZ, weight-for-height z-score; BMIZ, BMI-for-age z-score; IOTF, International Obesity Task Force; EC, Eastern Cape; KZN, KwaZulu-Natal; Limp, Limpopo; Mphu, Mphumalanga; NC, Northern Cape; NWP, North West Province; WC, Western Cape.

**Table 3:** Stratified analyses and assessment of the heterogeneity determinants for prevalence of combined overweight/obesity and for stunting

Overweight/obesity	Prs†	Pr	95 % CI	I^2^ (%)	I^2^_Res._ (%)	*P* value*
Strata: age 1–6 years						
Year‡	7	–		–	98·3	0·132
Rural area	3	18·8	0·0, 39·7	99·3	99·3	0·819
Urban area	1	12·4	6·9, 17·9	0·0		
Quality score‡	7	–		–	98·5	0·914
Random§	5	21·2	12·2, 30·0	98·7	98·4	0·281
Convenience§	2	8·4	1·2, 15·6	78·9		
Strata: age 7–14 years						
Year‡	12	–		–	95·7	0·109
Rural area	6	12·8	7·4, 18·1	97·6	97·1	0·985
Urban area	2	12·9	11·3, 14·5	0·0		
Quality score‡	12	–		–	96·7	0·439
Random§	8	13·3	9·0, 17·4	97·7	96·8	0·608
Convenience§	4	11·6	9·9, 13·3	48·3		
Strata: age 10–19 years						
Year‡	19	–		–	98·1	0·033
Rural area	3	12·8	7·4, 18·1	93·9	85·4	0·134
Urban area	6	18·1	14·9, 21·3	67·1		
Quality score‡	19	–		–	98·1	0·831
Random§	10	17·1	12·1, 22·0	99·2	98·5	0·150
Convenience§	9	21·6	18·0, 25·2	84·9		
Stunting						
Strata: age 0–6 years						
Year‡	42	–		–	96·9	0·234
Rural area	17	23·5	18·5, 28·3	95·9	97·1	0·108
Urban area	11	32·9	24·2, 41·3	98·1		
Quality score‡	42	–		–	97·3	0·198
Random§	11	22·8	17·7, 27·8	97·4	97·3	0·521
Convenience§	31	25·8	21·5, 29·9	97·3		
Strata: age 6–18 years						
Year‡	25	–		–	96·1	0·012
Rural area	10	10·2	6·1, 14·2	97·5	96·5	0·319
Urban area	6	13·6	9·6, 17·5	86·1		
Quality score‡	25	–		–	96·2	0·181
Random§	8	10·5	6·5, 14·4	98·1	95·3	0·642
Convenience§	17	11·5	9·6, 13·4	87·3		
Strata: age 13–18 years						
Year‡	6	–		–	89·5	0·658
Rural area	1	6·0	4·5, 7·6	–	–	0·551
Urban area	3	10·2	3·8, 16·5	87·2	87·2	
Quality score‡	6	–		–	89·2	0·777
Random§	2	9·3	2·9, 15·5	96·5	91·0	0·904
Convenience§	4	9·8	4·9, 14·5	80·9		

* *P* value of the regression model.

† Prs, number of estimates; I^2^_Res._ (%), residual I-square after regression.

‡ Variable considered as continuous.

§ Sampling (random *v.* convenience).

More infants younger than 9 months than older infants and young children were generally classified as overweight, with an apparent decrease in weight-for-length/height z-score (WHZ) from infancy up to the age of 59 months^(5,38,39)^. The best comparison can be made between results from different years of national surveys, using the same reference cut points for similar age groups. Based on the IOTF cut points, comparison of overweight and obesity prevalence between 1 to 6-year-old children from the National Food Consumption (NFCS) 2005^(36)^ and 2 to 5-year-old children from the South African National Health and Nutrition Examination Survey (SANHANES) 2012^(6)^ showed that overweight prevalence increased from a range of 12 to 16 % to 18·1 %, while obesity remained at a similar level. Based on the NCHS and WHO references, combined overweight and obesity prevalence below 10 % was reported in earlier studies^(23,40)^, while a prevalence above 10 % in most studies conducted after 2013^(6,41,42)^. The meta-analysis of prevalence data collected between 2005 and 2018 indicates that 18·9 % of children aged 1–6 years were overweight or obese (Fig. 3), and the meta-regression analysis showed no change in combined overweight and obesity over time (*P* = 0·13, Table 3). Publication bias was detected in the studies on stunting prevalence among children of 1–6 years old (*P* < 0·001), but not in studies reporting overweight and obesity prevalence in the same age group (*P* = 0·43).

#### Primary school-age children

The prevalence of stunting, overweight and obesity among primary school-aged children from national studies and selected regional studies with a large sample size is presented in Table 4.

**Table 4:** The prevalence of stunting, overweight and obesity among primary school-age children from national studies and selected regional studies with a large sample size by province and reference

Province study, setting	Year of study	Age group	Sample size	Stunted	Underweight	Overweight	Cut-point reference	Prevalence(%)	Reference
						Obese			
Reference: NCHS WHZ > 2/NCHS 85–95th									
National	1999	7–9 years	582	13·9	10·7	Overweight	NCHS WHZ > 2	5·8	Labadarios *et al.*, 2005^(35)^
Reference: CDC 2000 BMI > 85th and 95th percentiles (P)									
Limpopo RU	2007	9–13 years	602	8·8	4·0	Overweight	85–95th P	10·1	Malongane & Mbhenyane, 2017^(73)^
						Obese	> 95th p	0·8	
Limpopo R	2010	10–13 years	964	–	5·8	Overweight	85–95th p	10·8	Toriola *et al.*, 2015^(74)^
						Obese	> 95th p	5·2	
Limpopo Mphu RU	2017	9–13 years	1361	–	4·6	Overweight	85–95th p	10·2	Moselakgomo & Van Staden, 2017^(75)^
						Obese	> 95th p	5·4	
Limpopo R	2017	6–15 years	508	22·0	27·0	–	–	–	Modjadji & Madiba, 2019^(76)^
Reference: WHO 2007 BAZ									
WC RU	2008	10–12 years	717	19·3	2·0	Overweight	BAZ 1–2	14·3	Abrahams *et al.*, 2011^(45)^
						Obese	BAZ > 2	6·7	
WC RU	2009	9–13 years	1002	–	3·0	Overweight	BAZ 1–2	9·5	De Villiers *et al.*, 2016^(47)^
						Obese	BAZ > 2	18·6	
EC U	2015	8–12 years	801	–	4·9	Overweight	BAZ 1–2	13·2	Gerber *et al.*, 2018^(77)^
						Obese	BAZ > 2	5·1	
EC R	2015	6–12 years	1390	9·1	–	Overweight	BAZ > 1	14·9	Graham *et al.*, 2018^(78)^
EC U	2019	6–12 years	1277	9·1	–	–	–	–	Beckmann *et al.*, 2021^(79)^
Gauteng RU	2018	5–9 years	626	6·7	6·8	Overweight	BAZ 1–2	13·4	Senekal *et al.*, 2019^(42)^
						Obese	BAZ > 2	6·8	
International Obesity Task Force (IOTF) BMI cut points									
Mphu R	2007	5–9 years	970	5·0	6·0	Overweight	IOTF	4·0	Kimani-Murage *et al.*, 2010^(72)^
						Obese		1·0	
Mphu R	2007	10–14 years	944	7·0	7·0	Overweight	IOTF	6·0	Kimani-Murage *et al.*, 2010^(72)^
						Obese		2·0	
NWP RU	2000	10–15 years	1257	19·1	–	Overweight	IOTF	6·3	Mukuddem-Petersen & Kruger, 2004^(80)^
						Obese		1·6	
NWP U	2009	6–7 years	816	4·3	–	Overweight	IOTF	7·8	Kemp *et al.*, 2011^(81)^
						Obese		3·8	
KZN RU	1998	6–11 years	942	25·9	6·8	Overweight	IOTF	20·4	Jinabhai *et al.*, 2005^(71)^
						Obese		7·0	
KZN R	2010	7 years	514	–	3·9	Overweight	IOTF	9·9	Craig *et al.*, 2013^(56)^
						Obese		1·4	
KZN R	2010	11 years	503	–	3·4	Overweight	IOTF	8·5	Craig *et al.*, 2013^(56)^
						Obese		2·4	
KZN R	2011	7–14 years	959	9·2	9·6	Overweight	IOTF	9·0	Timaeus, 2012^(82)^
						Obese		3·8	
KZN RU	2012	7–11 years	1532	2·9	–	Overweight	IOTF	13·2	Houle *et al.*, 2016^(83)^
						Obese			
National	1999	7–9 years	544	13·0	10·7	Overweight	IOTF	6·5	Labadarios *et al.*,2005^(35)^
						Obese		3·0	
5 Provinces RU	2005	6–13 years	10 195	–	–	Overweight	IOTF	13·8	Armstrong *et al.*, 2006^(84)^
						Obese		3·5	
National	2012	7–9 years	929	9·4	8·6	Overweight	IOTF	8·3	Shisana *et al.*, 2014^(6)^
						Obese		3·4	
National	2012	10–14 years	1305	12·5	0	Overweight	IOTF	12·3	Shisana *et al.*, 2014^(6)^
						Obese		4·2	

R; rural, U, urban; M, male; F, female; NCHS, National Center for Health Statistics; HAZ, height-for-age z-score; WHZ, weight-for-height z-score; BAZ, BMI-for-age z-score; p, percentile; IOTF, International Obesity Task Force; EC, Eastern Cape; KZN, KwaZulu-Natal; Mphu, Mphumalanga; NWP, North West province; WC, Western Cape.

Complete data of the prevalence of stunting, overweight and obesity among primary school-aged children are presented in Table S2. A high prevalence of stunting was reported in studies in low socio-economic settings (19·3–30·8 %)^(43–45)^. The prevalence of stunting reported among children of 7–14 years old in nationally representative studies over time since 1999 was lower and remained at the same level between 13 % in 1999 and 12·5 % in 2012^(6,46)^. In these national studies, the prevalence of stunting was almost 50 % lower among children of 7–9 years old than in the 1–3-year-old group^(35,36)^. Meta-analysis of prevalence data shows a stunting prevalence of 11·3 % among primary school-age children based on studies published from 2006 to 2022 (Fig. 2). Meta-regression analysis shows a significant decrease in stunting over the time by a factor of 3·2 % (95 % CI = 0·9 %, 5·5 %) in studies including children 6–18 years old (*P* = 0·01), but not in the 13- to 18-year-old group (Table 3).

Children from low socio-economic status settings, in particular primary school-age boys^(43–45)^, had a higher prevalence of underweight than children from higher socio-economic status settings^(47,48)^. In national studies, there was an apparent decrease in underweight prevalence from 8 % in 1999 to 1·7 % in 2012^(6,35)^. Comparison of overweight and obesity prevalence between 7- to 9-year-old children from the NFCS 1999 (overweight 6·5 %, obesity 3 %) and the SANHANES 2012 (overweight 8·3 %, obesity 3·4 %) showed that overweight and obesity prevalence remained at similar levels^(6,35)^. The prevalence of combined overweight and obesity was higher than 20 % in several regional studies (21–28·7 %)^(42,45,47–50)^. Publication bias was detected in the studies on stunting prevalence among primary school-aged children (*P* < 0·001), but not in studies reporting overweight and obesity prevalence in the same age group (*P* = 0·87).

#### Adolescents

The prevalence of stunting, overweight and obesity among adolescents from national studies and selected regional studies with a large sample size is presented in Table 5, while complete data are presented in online Supplementary Table S3. A high prevalence of stunting was reported in studies in low socio-economic settings (16–30 %)^(31,51,52)^. The prevalence of stunting reported among adolescents in nationally representative studies over time showed no change from 2002 (11·4 %)^(53)^ to 2008 (13·1 %)^(7)^ and 2011 (12·9 %)^(54)^. In these national studies, the prevalence of stunting among adolescents was similar to the prevalence among children of 7–9 years old^(35,36)^. Adolescents from low socio-economic status settings^(31,55)^ had a higher prevalence of underweight than children from higher socio-economic status areas^(56–59)^. In national studies, the underweight prevalence did not change from 2002 to 2011 (9 % and 7 %, respectively)^(53,54)^. The prevalence of overweight increased among adolescents in the national surveys from 2002 (16·9 %, 95 % CI 14·3, 20·1 %) to 2011 (23·1 %, 95 % CI 21·5, 24·9 %), whereas the obesity prevalence remained unchanged (4 % and 6·9 %, respectively)^(7,54)^. The prevalence of combined overweight and obesity was higher than 20 % in several regional studies (21·3–42·3 %)^(51,57,58,60,61)^.

**Table 5:** The prevalence of stunting, underweight, overweight and obesity among adolescents from national studies and selected regional studies with a large sample size by province and reference

Province study setting	Year of study	Age group	Sample size	Stunted	Underweight	Overweight	Cut-point reference	Prevalence (%)	Reference
						Obese			
Reference: WHO 2006 WHZ/BAZ									
Gauteng U	2007	16–18 years	1172		–	Overweight	BAZ 1–2	12·6	Lundeen *et al.*, 2016^(85)^
						Obese	BAZ > 2	5·3	
Reference: CDC 2000 BMI > 85th and 95th percentiles									
KZN RU	2016	16–20 years	564	23·2	9·6	Overweight	85–95th ile	15·9	Bhimma *et al.*, 2018^(51)^
						Obese	> 95th ile	13·3	
International Obesity Task Force (IOTF) or WHO adult cut points									
Mphu R	2007	15–20 years	904	6·0	8·0	Overweight	IOTF	8·0	Kimani-Murage *et al.*, 2010^(72)^
						Obese		4·0	
KZN R	2013	15 years	502	–	3·4	Overweight	IOTF	13·1	Craig *et al.*, 2013^(56)^
						Obese		5·8	
WC U	2014	13–18 years	689	–	27·1	Overweight	IOTF	11·2	Van Niekerk *et al.*, 2013^(55)^
						Obese		4·8	
EC RU	2017	11–19 years	1360	–	8·5	Overweight	IOTF	17·5	Okeyo *et al.*, 2020^(64)^
						Obese		8·6	
National YRBS	2002	13–19 years	9442	11·4	9·0	Overweight	IOTF	16·9	Reddy *et al.*, 2009^(53)^
						Obese		4·0	
National DHS	2003	15–19 years	1256	–	20·5	Overweight	IOTF	12·2	DoH & MRC, 2007^(37)^
						Obese		4·0	
National YRBS	2008	13–19 years	9965	13·1	8·4	Overweight	IOTF	19·7	Reddy *et al.*, 2010^(7)^
						Obese		5·3	
National YRBS	2011	13–19 years	9816	12·9	7·0	Overweight	IOTF	23·1	Reddy *et al.*, 2013^(54)^
						Obese		6·9	
National DHS	2016	15–19 years	1043	2·1	13·4 (BMI < 18·5 kg/m^2^)	Overweight	BMI 25–30 kg/m^2^	10·7	NDoH, StatsSA & ICF, 2019^(5)^
						Obese	BMI > 30 kg/m^2^	6·6	

U, urban; R, rural; M, male; F, female; NCHS, National Center for Health Statistics; HAZ, height-for-age z-score; WHZ, weight-for-height z-score; BAZ, BMI-for-age z-score; EC, Eastern Cape; FS, Free State; KZN, KwaZulu-Natal; Mphu, Mphumalanga; NWP, North West province; WC, Western Cape; IOTF, International Obesity Task Force; ile, percentile; YRBS, Youth Risk Behaviour Survey; DHS, Demographic & Health Survey.

Meta-analysis of prevalence data shows a stunting prevalence of 9·6 % among adolescents based on the included studies (Fig. 3). Meta-regression analysis shows an increase in combined overweight and obesity over time in the 10–19 years age group (*P* = 0·03, Table 3). No publication bias was detected in the studies on stunting prevalence among adolescents (*P* = 0·65), or in studies reporting overweight and obesity prevalence in adolescents (*P* = 0·19).

## Discussion

Anthropometric data from population-based surveys can provide valuable information to track trends in the prevalence of malnutrition over time and to identify population groups who are at higher risk of malnutrition. This systematic review is characterised by a high degree of variability in indicators of malnutrition across age groups and provinces, which limits comparable across studies. Therefore, a meta-analysis of the prevalence data was conducted to show differences in prevalence between the age groups studied. The prevalence of overweight among preschool children was very low and seldom recorded before year 2000, particularly in rural areas^(23,40)^. Recently higher proportions of infants and preschool children from rural and urban settings were reported to be overweight^(6,62)^, but comparison with earlier data is difficult, because different reference cut points were used. The meta-analysis of prevalence data from studies that used the same cut points showed that similar proportions of preschool children and adolescents were overweight or obese (19 %), compared to 12·5 % of primary school-age children. An increase in overweight prevalence was observed among adolescents^(53,54)^. During adolescence, the higher prevalence of overweight and obesity among girls than boys become clear^(7,54)^. In terms of undernutrition, the prevalence of underweight and wasting at national level remained relatively low, but the prevalence of stunting was persistently high and remains unchanged among preschool children. Due to the high level of heterogeneity among studies (I^2^ 88·8–99·2 %), a meta-regression was conducted, and the results indicate a decrease in stunting prevalence among primary school-age children. The double burden of malnutrition is evident from this review, with stunting and overweight both reported among important proportions of children across the age range.

### Infants and preschool children

The higher prevalence of stunting among infants and young children (0–2-year-old) from low socio-economic areas^(22,27,30)^ compared to higher socio-economic urban areas^(19,28,31,38)^ is in line with the previous review^(8)^ and a recent review of global data^(86)^. The highest total under 5 years old prevalence of stunting, underweight and wasting was found among children from the Northern Cape province, where a large proportion of the population live in under-resourced settings^(33–35)^. The prevalence of stunting remained at similar levels among children younger and older than 5 years in nationally representative studies over time since 1999^(5,35–37)^. In most other regions, relatively low proportions of preschool children were underweight or wasted and the prevalence decreased further from 1999 to 2016^(5,35)^. A recent review of child undernutrition in low- and middle-income countries indicates that stunting prevalence among preschool children declined globally, but the reduction was the smallest in Africa^(86)^. The same review highlighted the association of maternal height and nutrition during pregnancy with infant birth length. This is an indication of intergenerational effects and continuing undernutrition in the offspring of mothers with chronic undernutrition. Experimental evidence of the beneficial effects of breast-feeding on early child growth is not consistent^(87)^, but a systematic review showed that complementary feeding interventions have a significant impact on linear growth of children in low- and middle-income countries^(88)^. Socio-economic drivers of undernutrition include low parental education, lack of household assets and limited access to clean water and sanitation^(89)^. These conditions are still present in low socio-economic status areas in South Africa^(33,36,43)^. A recent review of complementary feeding practices in South Africa revealed early introduction of foods and drinks with a low nutrient density, such as thin maize meal porridge, tea and sugar water^(90)^. The complementary feeding initiation period overlaps with the time when stunting prevalence increases in South Africa^(5)^.

These observations may indicate that complementary feeding interventions together with improved parental education and economic development may be useful to decrease the prevalence of stunting among preschool children in South Africa. Since 1997, several strategies of the South African government have been introduced to improve infant and young child nutrition, including the Infant and Young Child Feeding Policy (2007, revised 2013), the Tshwane Declaration of support for breastfeeding (2011), Regulations relating to Foodstuffs for Infants and young Children (2012), the Maternal, New-born, Child and Women’s Health and Nutrition Strategy (2012), the Roadmap for Nutrition in South Africa (2012), and the National Integrated Early Childhood Development Policy (2015)^(91)^. The latter centres around nurturing care for infants and young children, with nutrition as one of the five elements of the policy. Yet, the prevalence of stunting in children under-five remains consistently high.

The higher prevalence of overweight among preschool than primary school-age children is a cause of concern^(5,38,39)^, although observation of changes over time is difficult when the same reference cut points for similar age groups were not applied. The IOTF cut points were used to present overweight and obesity prevalence of 1–6-year-old children from the NFCS 2005^(36)^ and 2–5-year-old children from the SANHANES 201.^(6)^. Based on similar references, a combined overweight and obesity prevalence below 10 % was reported in earlier studies^(23,36,40)^, but the prevalence generally increased to more than 10 % in most recent studies^(6,41,42)^. The meta-analysis of the overweight and obesity prevalence data confirms the high prevalence of excess adiposity among South African children 1–6 years old. A longitudinal study in Johannesburg showed that girls who were overweight or obese at the age of 1–8 years had increased odds of being obese during late adolescence. Obesity was persistent among one-third of girls and 17 % of boys who became obese from the age of 1–2 years. Early childhood obesity should therefore not be ignored^(85)^.

### Primary school-age children

As expected, the highest prevalence of stunting and underweight was reported in studies in low socio-economic communities in both rural^(43)^ and urban settings^(44,45)^. The meta-regression results confirm findings from earlier studies that the prevalence of stunting among primary school-age children improved since 1999^(6,35,36)^ and was markedly lower than among 1–3-year-old children^(35,36)^, which may be an indication of some catch-up growth. In national studies, underweight prevalence appeared to decrease from 1999 to 2012^(5,35)^. Comparison of overweight and obesity prevalence among 7–9-year-old children from national surveys in 1999 (overweight 6·5 %, obesity 3 %) and 2012 (overweight 8·3 %, obesity 3·4 %) shows that overweight and obesity prevalence remained at similar levels^(6,35)^, but a high prevalence of combined overweight and obesity (20·2–24·1 %) was reported in several regional studies^(45,47,48,50,63)^.

Global reviews show increases in the prevalence of overweight and obesity among school-age children over time since 1975^(92)^. However, in general, obesity prevalence decreases from age 1 year up to the age of 14 years, with the lowest prevalence in the age group 10–14 years, and then increase throughout adolescence^(85)^. A longitudinal study in Johannesburg showed a low prevalence of overweight and obesity among school-age boys, declining from infancy throughout childhood, but obesity incidence was highest from age 4–8 years to 11–12 years in boys. The same increased incidence occurred later among girls, namely during early adolescence (from 11–12 years to 13–15 years), although overweight and obesity continued to increase throughout childhood in girls^(85)^. A recent study in the USA showed that the food and physical activity environment in primary schools is significantly associated with adiposity measures among the children in those schools. Food environment variables included unhealthy foods in school meals and vending machines, and physical activity environment included facilities for active play and sport participation^(93)^.

### Adolescents

A high prevalence of stunting and underweight was also reported among adolescents in studies in low socio-economic settings^(31,51,52)^. The prevalence of underweight among adolescents in nationally representative studies was at similar levels in 2002–2011^(53,54)^, and the prevalence of stunting was similar to the prevalence among children 7–9 years old^(35,36)^. The increasing prevalence of overweight from 16·9 % in 2002 to 23·1 % in 2011 in national studies^(53,54)^ was confirmed by the meta-regression results that showed an overall increase in overweight and obesity prevalence among adolescents. During adolescence, a significantly higher proportion of girls than boys were overweight and obese^(54)^. Regional studies show a high prevalence of overweight and obesity in most provinces in South Africa^(51,57,58,60,61,64)^. The nutrition transition in South Africa is associated with changes in lifestyle behaviours and an increasing prevalence of overweight and obesity^(94)^. Pradeilles et al.^(95)^ investigated the socio-economic factors associated with under- and overnutrition among South African adolescents. They reported different manifestations from the nutrition transition among male and female adolescents living in an urban area. A low wealth index was associated with a higher odd of being thin in boys, but a lower odd of being thin in girls. In the total group, caregiver education below tertiary education and the lowest tertile of wealth index were associated with a lower odd of being underweight.

Examples of strategies of the South African government to improve school-age child nutrition since 1997 include the Integrated School Health Policy (2012), in addition to the National School Nutrition Programme that has been introduced in 1994. Nutrition is one of the twelve issues covered in this policy^(96)^. The South African government has introduced numerous measures and policies aimed at improving the nutritional status of children of all age groups, including the iodisation of salt, fortification of staple foods with micronutrients, vitamin A supplementation and school feeding^(91,96)^. These factors may have contributed to a decrease in stunting among primary school-age children, but some programmes may have contributed to the higher overweight and obesity prevalence among preschool children and adolescents.

### Limitations of this review

A recent multi-survey study, which included South African height-for-age z-score (HAZ) and WHZ data for children of 0–59 months old, assessed the quality of anthropometric data from 145 Demographic and Health surveys (DHS). The assessment was done using a quality score that was based on completeness of the database, percentage implausible values, differences by month of birth and standard deviation of the HAZ and WHZ. Results showed that the quality of the anthropometric data varies between surveys, which may affect population-based estimates of malnutrition. The score for South African DHS data indicated low quality of data with a score at just above 0, compared to the higher scores for Peru and Guatemala of 1·5^(97)^. We excluded twenty-one publications considered for this review, based on a low-quality score due to small sample size and failure to use correct age-appropriate anthropometric cut points. We did not assess the quality of the data in more detail, and it is possible that some of the estimates may not be accurate.

### Conclusion and recommendations

The double burden of malnutrition with persistent stunting, underweight among primary school-age boys and obesity among infants and adolescent girls, is evident from the results of this review. Although underweight and wasting prevalence remained low, the increasing prevalence of overweight and obesity among infants, preschool children and adolescents is of concern. A recent comprehensive study with repeated measurements throughout childhood showed that high BMI during early childhood tends to stay high, and that normal BMI occasionally increases to high BMI, but the reverse is rarely true^(92)^. Early childhood and post-puberty appear to be important periods for intervention to prevent obesity, particularly among girls. Inadequate intakes of vegetables and fruit contribute to high energy density of diets, while low physical activity and the frequent consumption of unhealthy snacks and sugar-sweetened beverages are of concern^(54,60,65)^.

Limited success has been achieved in interventions targeting the double burden of malnutrition in children. Appropriate interventions proposed to curb the increase in obesity among children include to restrict advertisement of unhealthy foods to children, improving nutritional quality of school meals, tax on unhealthy foods and subsidies on healthy foods and supply chain incentives to produce more healthy foods^(98)^. An emphasis on appropriate interventions should always guide any food choices to avoid excessively processed foods and foods providing excessive amounts of nutrients of concern such as sugar, Na and saturated fat. More focused systematic reviews of the effects of interventions to improve infant and childhood anthropometric nutritional status will provide valuable information to guide childhood nutrition programmes and policies in South Africa and other low- and middle-income countries.

## Supplementary material

For supplementary material accompanying this paper visit https://doi.org/10.1017/S1368980023001994. click here to view supplementary material
