# Supplementary material for: Anthropometric nutritional status of children (0–18 years) in South Africa 1997–2022: a systematic review and meta-analysis
Source: Public Health Nutr. 2023 Oct 6;26(11):2226–42. doi: 10.1017/S1368980023001994 (PMC10641624; doi:10.1017/S1368980023001994)
Supplement: Supplementary file 1 [file S1368980023001994sup001.docx]

**Table S1. The prevalence of stunting, underweight, overweight and obesity among preschool children by province and reference**

| **Province**  **Study, setting** | **Year of study** | **Age group** | **Sample size, MF** | **Stunting** | **Under-weight** | **Overweight/ obesity** | **Cut-point reference** | **Prevalence**  **(%, 95%CI)**  **All/Male/**  **Female** | **Reference** |
| --- | --- | --- | --- | --- | --- | --- | --- | --- | --- |
|  |  | | **Reference: NCHS HAZ <-2; WHO2006 WAZ <-2 WHZ >2/ >85^th^ and 95^th^ percentiles** | | | | | | |
| Limpopo R | 1997 | 3-5y | 345 | - | - | Overweight/ obese | NCHS >85th | 1.7 | Monyeki *et al.,* 1999^(40)^ |
| KZN R | 1997 | 4mo-2y | 115 | 15.3 | 3.6 | Overweight | NCHS  WHZ >2 | 7.2 | Faber & Benadé, 1999^(21)^ |
| KZN R | 1998 | 6mo-2y | 97;  50M, 47F | 16.5 | 8.5 | Overweight | NCHS  WHZ >2 | 14.8 | Faber & Benadé, 2000^(99)^ |
| KZN R | 1999 | 2-5y | 164;  77M, 87F | 21.0 | 9.0 | Overweight | NCHS  WHZ >2 | 3.0 | Faber & Benadé*,* 2001^(21)^ |
| KZN U | 2007 | 3-9mo | 157 | 40.1 | 2.9 | Overweight |  | - | Pillay *et al*., 2021^(100)^ |
| National | 1999 | 1-3y | 1198 | 25.5 | 12.4 | Overweight | NCHS  WHZ >2 | 6.6 (5.2, 8.0) | Labadarios *et al,* 2000^(46)^ |
| WC U | 2000 | 0.5-1y | 113 | 1.8 | 0 | Overweight | NCHS  WHZ >2 | 35.0 | Sibeko *et al.,* 2004^(38)^ |
| NWP U | 2001 | 3-4y | 120;  58M, 62F | - | - | Overweight | BMI  85-95^th^ ile | 9.2 | Du Toit & Pienaar, 2003^(101)^ |
| NWP U | 2001 | 3-4y | 120;  58M, 62F | - | - | Obese | BMI >95^th^ ile | 6.7 | Du Toit & Pienaar, 2003^(101)^ |
| NWP U | 2014 | 1y | 392 | 39.9 | 13.6 | Overweight | WHO WHZ>2 | 5.1 | Rikhotso *et al*., 2022^(102)^ |
| EC R | 2003 | 0.5-2y | 767;  384M,383F | 10.7 | - | Overweight | NCHS  WHZ >2 | 15.2 | Smuts *et al.,* 2008^(65)^ |
| EC R | 2003 | 2-5y | 765;  383M,382F | - | - | Overweight | NCHS  WHZ >2 | 5.0 | Smuts *et al.,* 2008^(65)^ |
| Limpopo R | 2006 | <1y | 185;  99M, 86F | 18.9 | 7.0 | Overweight | NCHS WHZ>2 | 17.3 | Mushaphi *et al.,* 2008^(39)^ |
| Limpopo R | 2012 | <2y | 221 | 34.7 | 7.3 | Overweight |  | - | Maciel *et al*., 2021^(103)^ |
| Limpopo R | 2016 | <3y | 404 | 30.9 | 8.4 | Overweight | WHO WHZ>2 | N/A | Hill *et al.,* 2020^(104)^ |
| **Table S1. continuing** | | | **Reference: WHO 2006 HAZ <-2; WHZ/BAZ >2** | | | | | | |
| National | 1999 | 1-3y | 1160 | 25.5 | 12.4 | Overweight | WHZ >2 | 11.3 (9.3, 13.4) | Labadarios *et al*., 2000^(44)^ |
| National | 1999 | 4-6y | 993 | 23.4 | 11.0 | Overweight | WHZ >2 | 6.6 (4.6, 8.8) | Labadarios *et al*., 2000^(46)^ |
| KZN R | 2008 | <2y | 413 | 17.7 | 1.0 | Overweight | WHZ >2 | 8.7 | Schoeman *et al.,* 2010a^(22)^ |
| EC R | 2008 | <2y | 141 | 19.1 | 1.4 | Overweight | WHZ >2 | 6.4 | Schoeman *et al.,* 2010a^(22)^ |
| EC R | 2008 | 2-5y | 166 | 26.0 | 3.0 | Overweight | WHZ >2 | 1 | Schoeman *et al.,* 2010b^(105)^ |
| EC R | 2013 | <2y | 470 | 8.0 | 3.0 | Overweight |  | - | Stansert Katzen *et al.,* 2020^(106)^ |
| EC U | 2015-6 | <2y | 400;  199M,201F | 9.0 | - | Overweight | WHZ >2 | 16.0 | McLaren *et al*., 2018^(27)^ |
| KZN R | 2008 | 2-5y | 245 | 23.7 | 1.5 | Overweight | WHZ >2 | 0.9 | Schoeman *et al.,* 2010b^(105)^ |
| KZN U | 2016 | 4-6y | 627 | - | 12.0 | Overweight |  | - | Gruver *et al*., 2020^(66)^ |
| KZN RU | 2016 | 0-5y | 387 | 39.8 | 7.2 | Overweight  Obese | WHZ >2  WHZ >3 | 18.1  22.0 | Drysdale *et al.,* 2021^(107)^ |
| KZN R | 2018 | 0-5y | 567 | 14.0 | 1.9 | Overweight  Obese | WHZ 2-3  WHZ >3 | 11.8  3.7 | Gate *et al.,* 2020^(62)^ |
| KZN R | 2019 | 2-5y | 116 | 8.6 | - | Overweight  Obese | WHZ 2-3  WHZ >3 | 16.0  6.9 | Makanjana & Naicker 2020^(108)^ |
| WC R,U | 2009 | <5y | 179 | 10.7 | 8.5 | Overweight | WHZ >2 | 6.3 | Iversen *et al.,* 2011^(109)^ |
| KZN, WC, NC, Mphu RU | 2011 | 1-6y | 747 | 23.5 | 9.8 | Overweight | WHZ >2 | 6.1 | Faber *et al.,* 2015^(67)^ |
| WC R | 2012-5 | <1y | 1076 | - | - | Overweight | BAZ >2 | 9.0 | Budree *et al.,* 2017^(68)^ |
| Limpopo R | 2013 | 3-5y | 349;  136M,186F | 18.6 | 0.3 | Overweight | WHZ >2 | 20.9 | Motadi *et al.,* 2019^(41)^ |
| Limpopo U | 2019 | 0-5y | 415 | 45.3 | 29.0 | Overweight  Obese | WHZ 2-3  WHZ >3 | 8.2  3.7 | Modjadji & Madiba*,* 2019^(43)^ |
| NWP U | 2013-5 | 0.5y | 750;  387M,363F | 26.7 | 11.1 | Overweight | BAZ >2 | 10.1 | Matsungo *et al.,* 2017^(30)^ |
| NWP U | 2019 | 3-5y | 379 | 29.0 | 13.0 | Overweight | WHZ >2 | - | Modjadji & Madiba, 2019^(76)^ |
| Gauteng U | 2014 | 1-5y | 1254; 633M,621F | 35.8 | 20.5 | Overweight | BAZ >2 | 14.0 | Madiba *et al.,* 2019^(32)^ |
| Gauteng U | 2019 | 0-1y | 300 | 55.0 | 41.7 | Overweight  Obese | WHZ 2-3  WHZ >3 | 8.0  20.3 | Modjadji & Pitso, 2021^(110)^ |
| **Table S1. continuing** | |  |  |  |  |  |  |  |  |
| Gauteng U | 2019 | 3-5y | 102 | - | - | Overweight  Obese | WHZ 2-3  WHZ >3 | 2.9  1.0 | Soepnel *et al.,* 2021^(111)^ |
| NC U | 2017 | 6 weeks | 733 | 23.3 | 13.6 | Overweight | WHZ >2 | 11.0 | Le Roux *et al.,* 2020^(69)^ |
| National | 2016 | 1-5y | 1416; 721M,695F | 27.4 | 5.9 | Overweight | WHZ >2 | 13.3 | NDoH, StatsSA & ICF, 2019^(5)^ |
| National SA-NIDS | 2008 | 0.5y | 3254 | 11.0 | 5.2 | Overweight | WHZ >2 | 14.5 | Sartorius *et al*., 2020^(70)^ |
| Gauteng RU | 2018 | 0-5y | 674 | 21.6 | 5.6 | Overweight  Obese | BAZ 2-3  BAZ >3 | 10.3  7 | Senekal *et al.,* 2019^(42)^ |
|  |  | | **Reference: NCHS/WHO 2006 HAZ <-2; International Obesity Task Force (IOTF) cut-points** | | | | | | |
| KZN RU | 1998 | 2-5y | 770;  344M,426F | 33.7 | 5.8, 6.8 | Overweight | IOTF | 27.6, 23.9 | Jinabhai *et al.,* 2005^(71)^ |
| KZN RU | 1998 | 2-5y | 770;  344M,426F |  |  | Obese | IOTF | 13.9, 12.4 | Jinabhai *et al.,* 2005^(71)^ |
| National | 1999 | 1-3y | 795 | 25.5 | 12.5 | Overweight | IOTF | 16.0 (13.7,18.2) | Labadarios *et al.,* 2005^(35)^ |
| National | 1999 | 1-3y | 795 |  |  | Obese | IOTF | 7.8 (6.1, 9.5) | Labadarios *et al.,* 2005^(35)^ |
| National | 1999 | 4-6y | 861 | 20.7 | 8.8 | Overweight | IOTF | 12.0 (9.6, 14.4) | Labadarios *et al.*, 2005^(35)^ |
| National | 1999 | 4-6y | 861 |  |  | Obese | IOTF | 3.8 (2.5, 5.1) | Labadarios *et al.,* 2005^(35)^ |
| National | 2005 | 1-3y | 846 | 23.4 | 11.0 | Overweight | IOTF | 13.0 (10.8, 15.2) | Labadarios *et al.,* 2007^(36)^ |
| National | 2005 | 1-3y | 846 |  |  | Obese | IOTF | 6.3 (4.7, 7.8) | Labadarios *et al.,* 2007^(36)^ |
| National | 2005 | 4-6y | 745 | 20.7 | 8.8 | Overweight | IOTF | 8.3 (6.2, 10.4) | Labadarios *et al.,* 2007^(36)^ |
| National | 2005 | 4-6y | 745 |  |  | Obese | IOTF | 2.6 (1.4, 3.7) | Labadarios *et al.,* 2007^(36)^ |
| National | 2005 | 1-9y | 2157 | 18.0 |  | Overweight | IOTF | 10.0 (8.7, 11.3) | Labadarios *et al.,* 2007^(36)^ |
| National | 2005 | 1-9y | 2157 |  |  | Obese | IOTF | 4.0 (3.1, 4.8) | Labadarios *et al.,* 2007^(36)^ |
|  |  |  |  |  |  |  |  |  |  |
| **Table S1. continuing** | |  |  |  |  |  |  |  |  |
| Mphu R | 2007 | 1-4y | 671;  338M,333F | 18.0 | 10.0 | Overweight  Obese | IOTF | 7  1.0 | Kimani-Murage *et al.,* 2010^(72)^ |
| National | 2012 | 0-3y | 1090; 537M,553F | 26.5 | 8.2 | - | - | - | Shisana *et al.,* 2013^(6)^ |
| National | 2012 | 2-5y | 1291 | - | - | Overweight | IOTF | 18.1(11.4, 23.7) | Shisana *et al.,* 2013^(6)^ |
| National | 2012 | 2-5y | 1291 | - | - | Obese | IOTF | 4.6 (2.2, 7.0) | Shisana *et al.,* 2013^(6)^ |
| Mphu R | 2012 | 3-6y | 131 | 4.9 | 3.3 | Overweight  Obese | IOTF | 2.5  2.5 | Draper *et al.,* 2019^(112)^ |

U = urban, R = rural, M = male, F = female, NCHS = National Center for Health Statistics, HAZ = height-for-age z-score, WHZ = weight-for-height z-score, BAZ = body mass index-for-age z-score, EC = Eastern Cape, KZN = KwaZulu-Natal, Mphu = Mphumalanga, NC = Northern Cape, , NWP = North West Province, WC = Western Cape, IOTF = International Obesity Task Force

**Table S2. The prevalence of stunting, overweight and obesity among primary school-age children by province and reference**

| **Province**  **Study, setting** | **Year of study** | **Age group** | **Sample size** | **Stunted** | **Under-weight** | **Overweight**  **Obese** | **Cut-point reference** | **Prevalence**  **(%)** | **Reference** |
| --- | --- | --- | --- | --- | --- | --- | --- | --- | --- |
|  |  | | | | | **Reference: NCHS WHZ >2/** **NCHS 85-95^th^** | | | |
| National | 2005 | 7-9y | 582 | 13.9 | 10.7 | Overweight | NCHS WHZ>2 | 5.8 | Labadarios *et al.,* 2005^(35)^ |
|  |  | | | | | **Reference: CDC 2000 BMI >85^th^ and 95^th^ percentiles** | | | |
| KZN RU | 2004 | 2-12y | 1758 | 9.6 | 2.1 | Overweight  Obese | BAZ 1-2  BAZ >2 | 19.0  3.5 | Timaeus*,* 2012^(82)^ |
| Limpopo RU | 2007 | 9-13y | 602 | 8.8 | 4.0 | Overweight  Obese | 85-95^th^ p  >95^th^ p | 10.1  0.8 | Malongane & Mbhenyane, 2017^(73)^ |
| Limpopo R | 2010 | 10-13y | 964 | - | 5.8 | Overweight  Obese | 85-95^th^ p  >95^th^ p | 10.8  5.2 | Toriola *et al.,* 2015^(74)^ |
| Limpopo  Mphu RU | 2017 | 9-13y | 1361 | - | 4.6 | Overweight  Obese | 85-95^th^ p  >95^th^ p | 10.2  5.4 | Moselakgomo & Van Staden, 2017^(75)^ |
| Limpopo R | 2017 | 6-15y | 508 | 22.0 | 27.0 | - | - | - | Modjadji & Madiba, 2019^(43)^ |
|  |  | | | | | **Reference: WHO 2007 BAZ** | | | |
| NWP R | 2012 | 6-12y | 167 | 13.2 | 5.4 | Overweight  Obese | BAZ 1-2  BAZ >2 | 3.0  0.6 | Van der Hoeven *et al.,* 2016^(113)^ |
| WC RU | 2008 | 10-12y | 717 | 19.3 | 2.0 | Overweight  Obese | BAZ 1-2  BAZ >2 | 14.3  6.7 | Abrahams *et al.,* 2011^(45)^ |
| WC RU | 2009 | 9-13y | 1002 | - | 3.0 | Overweight  Obese | BAZ 1-2  BAZ >2 | 9.5  18.6 | De Villiers *et al.,* 2016^(47)^ |
| WC U | 2011 | 9-11y | 306 | - | - | Overweight | BAZ >2 | 26.1 | LeBlanc *et al.,* 2015^(48)^ |
| EC R | 2013 | 6-18y | 234 | 4.0 | 8.3 | Overweight  Obese | BAZ 1-2  BAZ >2 | 14.0  0 | Oldewage-Theron *et al*., 2017^(114)^ |
| EC U | 2015 | 8-12y | 801 | - | 4.9 | Overweight  Obese | BAZ 1-2  BAZ >2 | 13.2  5.1 | Gerber *et al.,* 2018^(77)^ |
| EC R | 2015 | 6-12y | 1390 | 9.1 | - | Overweight | BAZ >1 | 14.9 | Graham *et al.,* 2018^(78)^ |
| EC U | 2019 | 6-12y | 1277 | 9.1 | - | - | - | - | Beckmann et al. 2021^(79)^ |
| EC U | 2019 | 6-9y | 306 | - | - | Overweight/  Obese | BAZ >1 | 19.3 | Matjuda et al., 2020^(115)^ |
| KZN R | 2009 | 6-11y | 321 | 6.2 | 2.2 | Overweight  Obese | BAZ 1-2  BAZ >2 | 21.5  7.2 | Baumgartner *et al.,* 2012^(116)^ |
| **Table S2. Continuing** | |  |  |  |  |  |  |  |  |
| KZN R | 2011 | 7-14y | 959 | 9.2 | 4.0 | Overweight  Obese | BAZ 1-2  BAZ >2 | 9.0  3.8 | Tathiah *et al.,* 2013^(117)^ |
| KZN R | 2018 | 5-13y | 102 | 6.8 | 2.0 | Overweight  Obese | BAZ 1-2  BAZ >2 | 6.8  1.0 | Gate *et al*., 2020^(62)^ |
| Gauteng U | 2006 | 7-11y | 113 | 16.8 | 10.0 | Overweight  Obese | BAZ 1-2  BAZ >2 | 10.6  3.5 | Samuel *et al.,* 2010^(118)^ |
| Gauteng U | 2015 | 5-9y | 220 | 8.6 | 2.7 | Overweight | WHZ >2 | 24.1 | Shiau *et al.,* 2017^(49)^ |
| Gauteng RU | 2018 | 5-9y | 626 | 6.7 | 6.8 | Overweight  Obese | BAZ 1-2  BAZ >2 | 13.4  6.8 | Senekal *et al.,* 2019^(42)^ |
|  |  | | | | | **International Obesity Task Force (IOTF) BMI cut-points** | | | |
| Limpopo U | 2011 | 7-13y | 269 | - | - | Overweight | IOTF | 4.5 | Goon *et al.,* 2013^(119)^ |
| Mphu R | 2007 | 5-9y | 970 | 5.0 | 6.0 | Overweight  Obese | IOTF | 4.0  1.0 | Kimani-Murage *et al.,* 2010^(72)^ |
| Mphu R | 2007 | 10-14y | 944 | 7.0 | 7.0 | Overweight  Obese | IOTF | 6.0  2.0 | Kimani-Murage *et al.,* 2010^(72)^ |
| NWP RU | 2000 | 10-15y | 1257 | 19.1 | - | Overweight  Obese | IOTF | 6.3  1.6 | Mukuddem-Petersen & Kruger, 2004^(80)^ |
| NWP U | 2006 | 11-13y | 277 | - | - | Overweight  Obese | IOTF | 15.5  6.5 | Monyeki *et al.,* 2009^(50)^ |
| NWP U | 2009 | 6-7y | 816 | 4.3 | - | Overweight  Obese | IOTF | 7.8  3.8 | Kemp *et al.,* 2011^(81)^ |
| KZN RU | 1998 | 6-11y | 942 | 25.9 | 6.8 | Overweight  Obese | IOTF | 20.4  7.0 | Jinabhai *et al.,* 2005^(71)^ |
| KZN R | 2010 | 7y | 514 | - | 3.9 | Overweight  Obese | IOTF | 9.9  1.4 | Craig *et al.,* 2013^(56)^ |
| KZN R | 2010 | 11y | 503 | - | 3.4 | Overweight  Obese | IOTF | 8.5  2.4 | Craig *et al.,* 2013^(56)^ |
| KZN RU | 2012 | 7-11y | 1532 | 2.9 | - | Overweight | IOTF | 13.2 | Houle *et al.,* 2016^(83)^ |
| National | 1999 | 7-9y | 544 | 13.0 | 10.7 | Overweight  Obese | IOTF | 6.5  3.0 | Labadarios*,* 2000^(46)^ |
| 5 Provinces RU | 2005 | 6-13y | 10195 | - | - | Overweight  Obese | IOTF | 13.8  3.5 | Armstrong *et al.,* 2006^(84)^ |
| **Table S2. Continuing** | |  |  |  |  |  |  |  |  |
| National | 2012 | 7-9y | 929 | 9.4 | 8.6 | Overweight  Obese | IOTF | 8.3  3.4 | Shisana *et al.,* 2014^(6)^ |
| National | 2012 | 10-14y | 1305 | 12.5 | 0 | Overweight  Obese | IOTF | 12.3  4.2 | Shisana *et al.,* 2014^(6)^ |

R = rural, U = urban, M = male, F = female, NCHS = National Center for Health Statistics, CDC = Centers for Disease Control, p = percentile, HAZ = height-for-age z-score, WHZ = weight-for-height z-score, BAZ = body mass index-for-age z-score, EC = Eastern Cape, KZN = KwaZulu-Natal, Mphu = Mphumalanga, NWP = North West province, WC = Western Cape, IOTF = International Obesity Task Force

**Table S3. The prevalence of stunting, underweight, overweight and obesity among adolescents by province and reference**

| **Province**  **Study setting** | **Year of study** | **Age group** | **Sample size; M, F** | **Stunted** | **Under-weight** | **Overweight**  **Obese** | **Cut-point reference** | **Prevalence (%)** | **Reference** |
| --- | --- | --- | --- | --- | --- | --- | --- | --- | --- |
|  |  | | | | | **Reference: NCHS WHZ >2/ NCHS 85-95^th^ NCHS >95th** | | | |
| Limpopo RU | 1997 | 15y | 50 | 30.0 | 18.0 | - | - | - | MacIntyre *et al.,* 2006^(120)^ |
| WC U | 2000 | 15-18y | 60 F | - | 0 | Overweight  Obese | 85-95^th^ p  >95^th^ p | 19.7  2.2 | Caradas *et al.,* 2001^(121)^ |
|  |  | | | | | **Reference: CDC 2000 BMI >85^th^ and 95^th^ percentiles** | | | |
| Limpopo R | 2010 | 14-16y | 208;  122M, 86F | - | 8.0 | Overweight  Obese | 85-95^th^ p  >95^th^ p | 6.7  3.8 | Toriola *et al.,* 2015^(74)^ |
| EC U | 2015 | 13-17y | 388;  116M,276F | - | 1.0 | Overweight  Obese | 85-95^th^ p  >95^th^ p | 21.7  20.6 | Nkeh-Chungag *et al.,* 2015^(61)^ |
| KZN RU | 2016 | 16-20y | 564;  204M,360F | 23.2 | 9.6 | Overweight  Obese | 85-95^th^ p  >95^th^ p | 15.9  13.3 | Bhimma *et al.,* 2018^(122)^ |
|  |  | | | | | **Reference: WHO 2006 WHZ/BAZ** | | | |
| NWP U | 2004 | 12-16y | 313;  134M,179F | 16.3 | - | Overweight  Obese | BAZ 1-2  BAZ >2 | 7.3  1.3 | Mamabolo *et al.,* 2007^(27)^ |
| FS U | 2006 | 13-15y | 414;  174M,240F | 6.8 | 3.4 | Overweight  Obese | BAZ 1-2  BAZ >2 | 16.2  6.0 | Meko *et al.,* 2015^(58)^ |
| Gauteng U | 2007 | 16-18y | 1172;  566M,606F |  | - | Overweight  Obese | BAZ 1-2  BAZ >2 | 12.6  5.3 | Lundeen *et al.,* 2016^(85)^ |
| EC R | 2013 | 14-18y | 98;  48M, 50F | 8.2 | 2.1 | Overweight  Obese | BAZ 1-2  BAZ >2 | 15.3  2.0 | Oldewage-Theron *et al*., 2014^(59)^ |
| Gauteng U | 2014 | 13-18y | 136 | - | 3.7 | Overweight  Obese | BAZ 1-2  BAZ >2 | 13.2  8.1 | McVeigh & Meiring, 2014^(123)^ |
| KZN R | 2018 | 14-19y | 31 | - | 3.2 | Overweight  Obese | BAZ 1-2  BAZ >2 | 32.2  3.2 | Gate *et al.,* 2020^(62)^ |
|  |  | | | | | **International Obesity Task Force (IOTF) cut-points** | | | |
| WC U | 2005 | 14-16y | 166;  64M, 102F | - | - | Overweight  Obese | IOTF | 19.3  4.2 | Somers *et al.,* 2006^(60)^ |
| Mphu | 2007 | 15-20y | 904; 432M,472F | 6.0 | 8.0 | Overweight  Obese | IOTF | 8.0  4.0 | Kimani-Murage *et al.,* 2010^(72)^ |
| **Table S3. Continuing** | |  |  |  |  |  |  |  |  |
| KZN R | 2013 | 15y | 502;  182M,320F | - | 3.4 | Overweight  Obese | IOTF | 13.1  5.8 | Craig *et al.,* 2013^(56)^ |
| WC U | 2014 | 13-18y | 689;  342M,347F | - | 27.1 | Overweight  Obese | IOTF | 11.2  4.8 | Van Niekerk *et al.,* 2013^(55)^ |
| NWP U | 2011 | 15y | 186 | - | 17 | Overweight/  Obese | IOTF | 13.0 | Masocha *et al*., 2020^(124)^ |
| NWP RU | 2016 | 11-13y | 381 | - | - | Overweight/  Obese | IOTF | 18.9 | De Waal & Pienaar, 2021^(20)^ |
| EC RU | 2016 | 10-14y | 411 | - | - | Overweight  Obese | IOTF | 9.3,  13.4 | Chungag *et al*., 2021^(125)^ |
| EC RU | 2017 | 11-19y | 1360 | - | 8.5 | Overweight/  Obese | IOTF | 17.5  8.6 | Okeyo *et al*., 2020^(64)^ |
| EC U | 2019 | 13-16y | 244 | - | 4.1 | Overweight/  Obese | IOTF | 16.8  15.5 | Letswalo *et al*., 2021^(126)^ |
| National RU YRBS | 2002 | 13-19y | 9442;  4870M, 4572F | 11.4 | 9.0 | Overweight  Obese | IOTF | 16.9  4.0 | Reddy *et al.,* 2009^(53)^ |
| National RU DHS | 2003 | 15-19y | 1256;  625M,634F | - | 20.5 | Overweight  Obese | IOTF | 12.2  4.0 | DoH & MRC, 2007^(37)^ |
| National RU YRBS | 2008 | 13-19y | 9965;  4870M,  5058F | 13.1 | 8.4 | Overweight  Obese | IOTF | 19.7  5.3 | Reddy *et al.,* 2009^(53)^ |
| National RU YRBS | 2011 | 13-19y | 9816;  4614M,  5202F | 12.9 | 7.0 | Overweight  Obese | IOTF | 23.1  6.9 | Reddy *et al.,* 2013^(54)^ |
| National RU DHS | 2016 | 15-19y | 1043;  499M,544F | 2.1 | 13.4 | Overweight  Obese | WHO adult BMI | 10.7  6.6 | NDoH, StatsSA & ICF, 2019^(5)^ |

U = urban, R = rural, M = male, F = female, NCHS = National Center for Health Statistics, CDC = Centers for Disease Control, p = percentile, HAZ = height-for-age z-score, WHZ = weight-for-height z-score, BMIZ = body mass index-for-age z-score, EC = Eastern Cape, FS = Free State, KZN = KwaZulu-Natal, Mphu = Mphumalanga, NWP = North West province, WC = Western Cape, YRBS = Youth Risk Behaviour Survey, DHS = Demographic & Health Survey, IOTF = International Obesity Task Force

**Additional References for Supplementary Tables**

99. Faber M & Benade AJ (2000) Factors associated with low serum retinol levels in children aged 6±24 months in a rural South African community. *Public Health Nutr* **3**, 395-402.

100. Pillay L, Moodley D, Emel LM *et al.* (2021) Growth patterns and clinical outcomes in association with breastfeeding duration in HIV exposed and unexposed infants: a cohort study in KwaZulu Natal, South Africa. *BMC Pediatr* **21**, 183.

101. Du Toit D, Pienaar AE (2003) Overweight and obesity and motor proficiency of 3- and 4-year old children. *South African journal for research in sport, physical education and recreation* **25**, 37-48.

102. Rikhotso IP, Faber M, Rothman M *et al.* (2022) Nutritional status and psychomotor development in 12–18-month-old children in a post-intervention study. *S Afr J Clin Nutr* **35**, 69-77.

103. Maciel BLL, Costa PN, Filho JQ *et al.* (2021) Higher energy and zinc intakes from complementary feeding are associated with decreased risk of undernutrition in children from South America, Africa, and Asia. *J Nutr* **151**, 170-178.

104. Hill CL, McCain K, Nyathi ME *et al.* (2020) Impact of Low-Cost Point-of-Use Water Treatment Technologies on Enteric Infections and Growth among Children in Limpopo, South Africa. *Am J Trop Med Hyg* **103**, 1405-1415.

105. Schoeman S, Smuts CM, Faber M *et al.* (2010b) Primary health care facility infrastructure and services and the nutritional status of children 0 to 71 months old and their caregivers attending these facilities in four rural districts in the Eastern Cape and KwaZulu-Natal provinces, South Africa. *S Afr J Clin Nutr* **23**, 21-27.

106. Stansert Katzen L, Tomlinson M, Christodoulou J *et al.* (2020) Home visits by community health workers in rural South Africa have a limited, but important impact on maternal and child health in the first two years of life. *BMC Health Serv Res* **20**, 594.

107. Drysdale RE, Bob U & Moshabela M (2021) Coping through a drought: the association between child nutritional status and household food insecurity in the district of iLembe, South Africa. *Public Health Nutr* **24**, 1052-1065.

108. Makanjana O & Naicker A (2020) Nutritional Status of Children 24-60 Months Attending Early Child Development Centres in a Semi-Rural Community in South Africa. *Int J Environ Res Public Health* **18**.

109. Iversen PO, Hoisaether EA, Morseth M *et al.* (2011) Diverging opinions of supplementation programmes between mothers of small children and staff at primary health clinics in the Western Cape Province of South Africa. *Public Health Nutr* **14**, 923-930.

110. Modjadji P & Pitso M (2021) Maternal Tobacco and Alcohol Use in Relation to Child Malnutrition in Gauteng, South Africa: A Retrospective Analysis. *Children (Basel)* **8**.

111. Soepnel LM, Nicolaou V, Slater C *et al.* (2021) Obesity and adiposity of 3- to 6-year-old children born to mothers with hyperglycaemia first detected in pregnancy in an urban South African setting. *Ann Hum Biol* **48**, 81-92.

112. Draper CE, Tomaz SA, Jones RA *et al.* (2019) Cross-sectional associations of physical activity and gross motor proficiency with adiposity in South African children of pre-school age. *Public Health Nutr* **22**, 614-623.

113. van der Hoeven M, Faber M, Osei J *et al.* (2016) Effect of African leafy vegetables on the micronutrient status of mildly deficient farm-school children in South Africa: a randomized controlled study. *Public Health Nutr* **19**, 935-945.

114. Oldewage-Theron W & Kruger R (2017) The association between diet quality and subclinical inflammation among children aged 6-18 years in the Eastern Cape, South Africa. *Public Health Nutr* **20**, 102-111.

115. Matjuda EN, Engwa GA, Letswalo PB *et al.* (2020) Association of hypertension and obesity with risk factors of cardiovascular diseases in children aged 6-9 years old in the Eastern Cape Province of South Africa. *Children (Basel)* **7**.

116. Baumgartner J, Smuts CM, Malan L *et al.* (2012) Effects of iron and n-3 fatty acid supplementation, alone and in combination, on cognition in school children: a randomized, double-blind, placebo-controlled intervention in South Africa. *Am J Clin Nutr* **96**, 1327-1338.

117. Tathiah N, Moodley I, Mubaiwa V *et al.* (2013) South Africa's nutritional transition: overweight, obesity, underweight and stunting in female primary school learners in rural KwaZulu-Natal, South Africa. *S Afr Med J* **103**, 718-723.

118. Samuel FO, Egal AA, Oldewage-Theron WH *et al.* (2010) Prevalence of zinc deficiency among primary school children in a poor peri-urban informal settlement in South Africa. *Health SA Gesondheid* **15**.

119. Goon D, Amusa L, Mhlongo D *et al.* (2013) Elevated Blood Pressure among Rural South African Children in Thohoyandou, South Africa. *Iran J Public Health* **42**, 489-496.

120. Macintyre UE, Du PLessis JB (2006) Dietary intakes and caries experience in children in Limpopo Province, South Africa. *S Afr Dent J* **61**, 58-63.

121. Caradas AA, Lambert EV & Charlton KE (2001) An ethnic comparison of eating attitudes and associated body image concerns in adolescent South African schoolgirls. *J Hum Nutr Diet* **14**, 111-120.

122. Bhimma R, Naicker E, Gounden V *et al.* (2018) Prevalence of Primary Hypertension and Risk Factors in Grade XII Learners in KwaZulu-Natal, South Africa. *Int J Hypertens* **2018**, 3848591.

123. McVeigh J & Meiring R (2014) Physical Activity and Sedentary Behavior in an Ethnically Diverse Group of South African School Children. *J Sport Sci Med* **13**, 371-378.

124. Masocha V, Monyeki MA & Czyz SH (2020) Longitudinal relationships between changes in body composition and changes in selected metabolic risk factors (abdominal obesity and blood pressure) among South African adolescents. *Peer J* **8**, e9331.

125. Chungag A, Engwa GA, Sewani-Rusike CR *et al.* (2021) Effect of seasonal variation on the relationship of indoor air particulate matter with measures of obesity and blood pressure in children. *J Health Pollut* **11**, 210610.

126. Letswalo BP, Schmid-Zalaudek K, Brix B *et al.* (2021) Cardiometabolic risk factors and early indicators of vascular dysfunction: a cross-sectional cohort study in South African adolescents. *BMJ Open* **11**, e042955.
